# Modeling demyelination and endogenous remyelination in spinal cord *ex vivo* rat organotypic slice cultures

**DOI:** 10.3389/fncel.2024.1345042

**Published:** 2024-06-26

**Authors:** Brooke Hawker, Muna Dhakal, Bronwen Connor, Amy McCaughey-Chapman

**Affiliations:** Department of Pharmacology and Clinical Pharmacology, Centre for Brain Research, School of Medical Science, Faculty of Medical and Health Sciences, University of Auckland, Auckland, New Zealand

**Keywords:** demyelination, slice culture, LPS, LPC, longitudinal spinal cord slices, multiple sclerosis, spinal cord injury

## Abstract

**Introduction:**

Demyelination of the spinal cord is a prominent feature of multiple sclerosis (MS) and spinal cord injuries (SCI), where impaired neuronal communication between the brain and periphery has devastating consequences on neurological function. Demyelination precedes remyelination, an endogenous process in which oligodendrocyte precursor cells (OPCs) differentiate into mature, myelinating oligodendrocytes with the ability to restore the myelin sheath and reinstate functional nerve signaling. However, in MS or SCI, demyelination is more severe, persistent, and inhibitory to OPC-mediated remyelination, leading to a permanent loss of neuronal function. Currently, there are no effective treatments for demyelination, and existing pre-clinical models typically focus on brain tissue with little characterization of demyelination within the spinal cord. Organotypic slice cultures are a useful tool to study neurological disease, providing a more complex 3-dimensional system than standard 2-dimensional in vitro cell cultures.

**Methods:**

Building on our previously developed rat brain slice culture protocol, we have extended our findings to develop a rat longitudinal spinal cord ex vivo model of demyelination.

**Results:**

We generated rat longitudinal spinal cord slice cultures that remain viable for up to 6 weeks in culture and retain key anatomical features of the spinal cord’s cytoarchitecture. We show that treating longitudinal spinal cord slices with lysolecithin (LPC) induced robust demyelination with some endogenous remyelination, which was not seen following exposure to lipopolysaccharide (LPS).

**Discussion:**

Our ex vivo organotypic spinal cord slice culture system provides a platform to model demyelination and endogenous remyelination long-term, mimicking that observed in LPC-induced rodent models of demyelination. This platform is suitable for the development and testing of novel therapeutic strategies with ease of manipulation prior to in vivo experimentation.

## Introduction

The spinal cord is the information relay center of the central nervous system (CNS), a critical piece of nervous tissue extending from the base of the brain stem down through the spinal column, facilitating communication between the brain and the periphery (Silva et al., 2014). Internally, the spinal cord is made up of central gray matter, a region composed of cell bodies, myelinated and non-myelinated axons, glia, and capillaries, all of which is surrounded by a region of white matter, composed of oligodendrocytes, astrocytes, and microglia (Ahuja et al., 2017; Zhou et al., 2019). Oligodendrocytes (OLs) are myelinating cells of the CNS, known for their role in producing the lipid-rich protein myelin that wraps around neuronal axons, forming the myelin sheath. The myelin sheath is critical for neuronal function, providing physical, metabolic, and trophic support while also acting as an insulating shield facilitating rapid saltatory action potential propagation of nerve signals (Bradl and Lassmann, 2010).

Demyelination refers to the loss of OLs and the myelin sheath, a process that strips neurons of this supportive coating, impairing nerve signal transmission while also leaving neurons exposed and vulnerable to damage (Alizadeh et al., 2015). MS is an autoimmune-mediated demyelinating disorder in which OLs and the myelin sheath targeted and destroyed by the immune system, generating focal lesions of demyelination seen in both the brain and spinal cord (Thompson et al., 2018). Similarly, demyelination occurs upon injury to the CNS, such as in spinal cord injuries (SCI), where following the initial injury, a cascade of biochemical events ensues, generating a highly inflammatory environment causing death to OLs, stripping surviving axons of the myelin sheath and impairing neuronal function within the site of injury (Almad et al., 2011). Demyelination within the cord blocks the critical information relay between the brain and periphery, causing neurological impairment below the site of demyelination.

In the healthy CNS, demyelination is followed by remyelination, an endogenous process in which quiescent oligodendrocyte precursor cells (OPCs) become activated, proliferate, and differentiate into mature, myelinating OLs with the ability to restore the myelin sheath and reinstate functional nerve signaling (Bradl and Lassmann, 2010). However, in the case of MS or SCI, demyelination is more severe, persistent, and inhibitory to OPC-mediated remyelination, rendering this process ineffective. Instead, incomplete remyelination is observed where the myelin sheath and neuronal function is only partially restored (Franklin, 2002; Alizadeh et al., 2015).

The generation of effective demyelination models is critical to the development of therapies for MS and SCIs however, traditional models tend to focus solely on demyelination within the brain. Spinal cord demyelination models have been developed, typically involving the generation of focal regions of demyelination via direct injection of chemical agents into the spinal cord such as lysolecithin (LPC), lipopolysaccharide (LPS) or ethidium bromide (EtBr) (Hall, 1972; Blakemore, 1982; Chu et al., 2019). These *in vivo* models have been invaluable in investigating demyelination within the spinal cord however, they are costly, time-consuming, and technically challenging. The development of *ex vivo* organotypic slice cultures (OSCs) has provided a platform to bridge the gap between *in vitro* cell culture models and *in vivo* animal models. OSCs are slices of tissue cultured in a way that preserves the 3-dimensional cytoarchitecture, anatomical organization, and retains cellular interactions between neighboring cells and proteins of the extracellular matrix (Pandamooz et al., 2016). Initially developed by Gähwiler (1981) using the roller-tube method, OSCs have now become a standard pre-clinical model cultured using the air-liquid interface method developed by Stoppini et al. (1991). This allows slices to receive nutrients from culture media and humidified air at the same time, enabling long-term culture which is critical for disease modeling. The age of the animals used is also an important factor in enabling long-term culture. The standard is to prepare slices from p9-p11 animals who display sufficient endogenous myelination, a factor critical when studying demyelination (Birgbauer et al., 2004; Humpel, 2015).

A variety of CNS tissues can be cultured *ex vivo,* with studies reporting the successful culture of hippocampal, cerebellar, cortical, and spinal cord tissue (Skrede and Westgaard, 1971; Llinás and Sugimori, 1980; Götz and Bolz, 1992; Bonnici and Kapfhammer, 2008). These cultures can be used to model various aspects of injury, including excitotoxicity (Guzman-Lenis et al., 2009; McCaughey-Chapman and Connor, 2017, 2022), inflammation (Felts et al., 2005; Cammarota and Boscia, 2023) and demyelination (Birgbauer et al., 2004; Cho et al., 2012). The use of OSCs to model demyelination is a relatively well-established process, achieved through treating slices with the glial toxin LPC at 0.5 mg/mL for 17 h (Birgbauer et al., 2004) or 15 μg/mL LPS for 24 h (Di Penta et al., 2013). However, these models usecerebellar tissue failing to characterize demyelination of the spinal cord in an *ex vivo* system. Spinal cord OSCs have been developed however, are typically generated through transverse sectioning, a process that represents the cord at a single region and severs axonal projections through spinal levels (Sypecka et al., 2015). To overcome this, Bonnici and Kapfhammer (2008) generated longitudinal spinal cord OSCs that incorporate multiple spinal levels within a single culture as well as retain axonal projections, a factor critical when considering myelination along the length of the cord.

The current study builds on our established rat brain sagittal organotypic slice culture system which we have used to model the cellular pathology involved in various diseases including Parkinson’s and Huntington’s disease (McCaughey-Chapman and Connor, 2017, 2022). We now extend these findings to model demyelination in rat longitudinal spinal cord slice cultures. We show that longitudinal spinal cord slices remain viable for up to 6 weeks in culture with retention of the gray matter/white matter boundary. We compare the effect of treatment with LPC versus LPS on demyelination and endogenous remyelination within the spinal cord slices. Overall, this study shows that rat longitudinal spinal cord slice cultures can be used as long-term *ex vivo* models of demyelination and endogenous remyelination relevant in MS and SCI.

## Materials and methods

### Animals

Postnatal 9- to 11-day old male Sprague–Dawley rat pups were used in this study. All animals were housed in a 12-h light–dark cycle with access to food and water *ad libitum*. Animal euthanasia was in accordance with the New Zealand Animal Welfare Act 1999 and was approved by the University of Auckland Animal Ethics Committee. All efforts were made to minimize the number of animals used.

### Rat longitudinal spinal cord organotypic slice culture generation and culturing

To generate longitudinal spinal cord slices we adapted our protocol for sagittal brain organotypic slice cultures (McCaughey-Chapman and Connor, 2017; McCaughey-Chapman and Connor, 2022). To expose the spinal cord, skin was removed from the neck to tail of the animal. The spinal cord was isolated through cutting through the vertebral column on both sides of the cord, which allowed the column to be peeled away. Using tweezers, the cord was carefully removed at the greatest length possible and mounted whole onto a vibratome chuck [Leica Biosystems] with superglue. The cord was sectioned in the longitudinal plane at 300 μm thickness, each cord generating 4–5 slices. During sectioning the vibratome chamber was filled with ice-cold medium consisting of Advanced DMEM/F-12 with 1% penicillin–streptomycin [Thermo Fisher Scientific, #12634010 and #15140148]. Individual slices were mounted onto sterile membrane inserts in 6-well plates [Corning, #COR3450] and cultured at the air-membrane interface at 35°C with 5% CO_2_. Slices were cultured in MEM with Hanks balanced salts [Thermo Fisher Scientific, #11575032], 1% penicillin–streptomycin and 25% horse serum [Thermo Fisher Scientific, #16050130] for 3 days (1 mL of medium added below the membrane insert). To limit glial scar formation, a cocktail of three mitotic inhibitors was added to the medium for the first three days of culturing: uridine, 5-fluorodeoxyuridine and cytosine-ß-arabinofuranoside [4.4 mM each, Sigma, #U3003, #F0503 and #C1768]. The slices were then transitioned into a serum-free medium consisting of Advanced DMEM/F-12 with 2% B-27 supplement [Thermo Fisher Scientific, #17504044] and 1% N-2 supplement [Thermo Fisher Scientific, #17502048] and cultured for up to 6 weeks.

### LPC and LPS treatment

**Figure 1:**
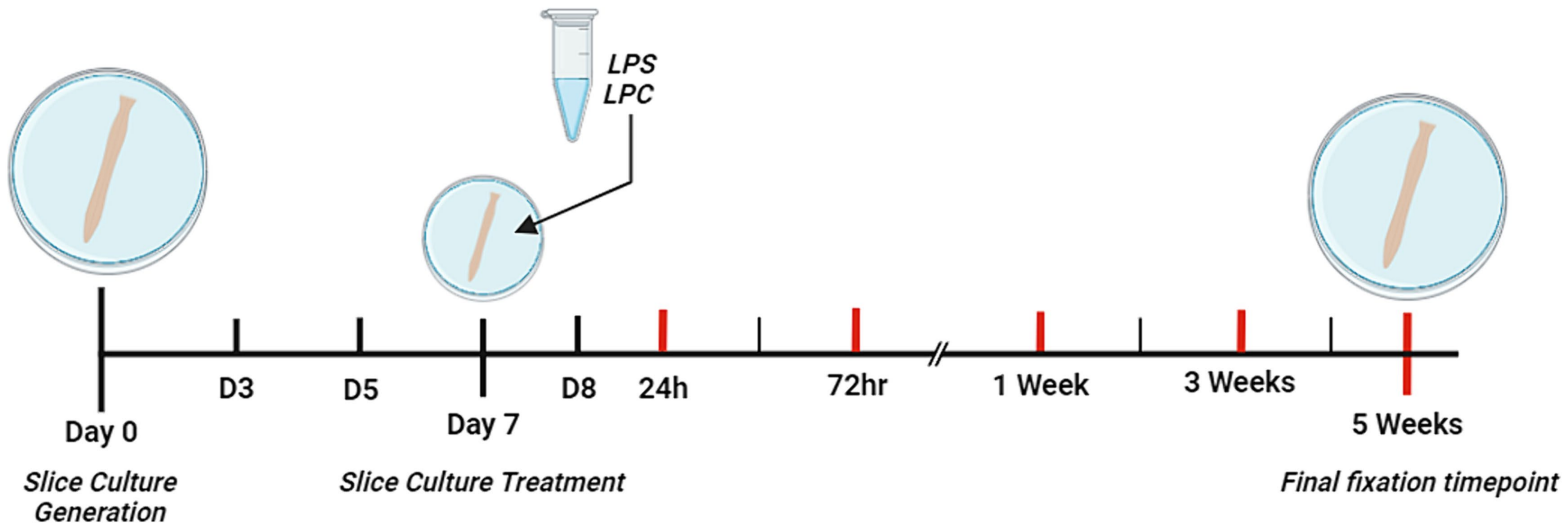
Schematic diagram depicting the timeline of the study. *Ex vivo* organotypic spinal cord slices were generated from p9 – p11 male, Sprague–Dawley rat pups. From Day 0 to Day 3 (D3) of culture, slices were cultured in the presence of three mitotic inhibitors (Cytosine-β-D-arabinofuranoside, Uridine, 5-Fluoro-2′-deoxyuridine) each at a concentration of 4.4 mM. On Day 7 of culture, slices were treated with either 15 μg/mL LPS for 24 h, 0.5 mg/mL LPC for 17 h or left untreated. Red lines indicate the 5 different timepoints post-treatment at which slices were fixed for analysis (24 h, 72 h, 1 week, 3 weeks and 5 weeks). Created with BioRender.com.

At Day 7 of culturing, slices were divided into three groups: untreated, LPS-treated or LPC-treated. Slices were cultured for 7 days prior to treatment with LPS [Sigma, #L2630] or LPC [Sigma, #62962] to allow for complete myelination of the tissue (Birgbauer et al., 2004). To induce demyelination, the slices were incubated for 24 h in 15 μg/mL LPS based on the study by Di Penta et al. (2013), or incubated for 17 h in 0.5 mg/mL LPC based on the study by Birgbauer et al. (2004). Thereafter slices were cultured for an additional 24 h, 72 h, 1, 3, or 5 weeks, in serum-free culture medium. A timeline for the study is summarized in Figure 1.

### Calcein-AM and ethidium homodimer-1 live tissue staining

Cell viability was assessed throughout untreated longitudinal spinal cord slices weekly up to 6 weeks in culture. To determine the effect of treatment with LPS or LPC on slice culture viability, cell viability was assessed in untreated, LPS-treated and LPC-treated slices at 24 h, 72 h, 1 week, 3 weeks and 5 weeks post-treatment (*n* = 4 slices per time point and per treatment). Cell viability was measured by live tissue staining with the live cell marker, calcein-AM [Thermo Fisher Scientific, #C1430], and the dead cell marker, ethidium homodimer-1 (EthD-1) [Thermo Fisher Scientific. #E1169]. As previously described, the slices were incubated in 2 μM calcein AM and 4 μM EthD-1 for 40 min at room temperature, at the desired time point, then rinsed with PBS and immediately imaged to avoid fluorescent signal degradation (McCaughey-Chapman and Connor, 2017).

### Immunohistochemistry

Slices were fixed in 4% paraformaldehyde for 24 h at 4°C. Whole mount slices were stained using a shortened version of the optical clearing technique iDISCO (Renier et al., 2014), as previously described (McCaughey-Chapman and Connor, 2022). Briefly, after 24 h of permeabilization, the slices were blocked and then incubated in primary antibody for 48 h. The extent of demyelination and remyelination was assessed by quantification of the expression of Myelin Basic Protein (MBP) [1:250, MAB386] and Myelin Oligodendrocyte Glycoprotein (MOG) [1:250, ab233549] (*n* = 3 slices per time point and treatment for each MBP and MOG). Dendritic sparing was qualitatively assessed by the expression of Microtubule-associated Protein 2 (MAP2) [1:500, ab92434]. The presence of astrocytes in cultures was qualitatively assessed by the expression of Glial Fibrillary Acidic Protein (GFAP) [1:250, G3893]. Alexa Fluor conjugated secondary antibodies were used to fluorescently label the antigens of interest and following dehydration in a series of increasing concentrations of methanol and clearing in dibenzyl ether, the membrane-bound slices were placed onto a glass microscope slide and imaged using a Nikon TE2000E inverted fluorescent microscope, Zeiss LSM 710 inverted confocal microscope and Zeiss LSM 800 inverted confocal microscope.

### Quantification and statistical analysis

Four images were captured throughout the white matter region of the spinal cord slices for all stains, with each of the four images capturing a similar area across slices. The ratio of calcein AM+/EthD-1+ cells was quantified using a pixel intensity macro in ImageJ, following consistent thresholding across images and background subtraction. The data was reported as the average percentage viability for each time point and treatment (*n* = 4 slices). The extent of MBP, MOG and GFAP staining was assessed by measuring the integrated density in ImageJ, following consistent background subtraction and threshold adjustment across all images. The total MBP+, MOG+ and GFAP+ density was calculated for each slice. An average per timepoint and treatment group was calculated (*n* = 3 slices). The MBP+ and MOG+ fluorescence data was reported as a percentage of the fluorescence intensity in 24-h untreated slices. The GFAP+ fluorescence data was reported as a percentage of the fluorescence intensity in 1-week untreated slices. GFAP+ cell process analysis was undertaken by capturing images of GFAP+ astrocytes in spinal cord slices, converting them to 8-bit and with NeuronJ [Java] manually measuring the number of processes, total process length and number of branches. Statistical significance was determined using raw values and a one-way ANOVA for the viability data in untreated spinal cord slices, and a two-way ANOVA for all other datasets, followed by post-hoc analysis using Tukey’s post-hoc test in the case of a significant ANOVA.

## Results

### Myelination occurs in the longitudinal spinal cord slice cultures

**Figure 2:**
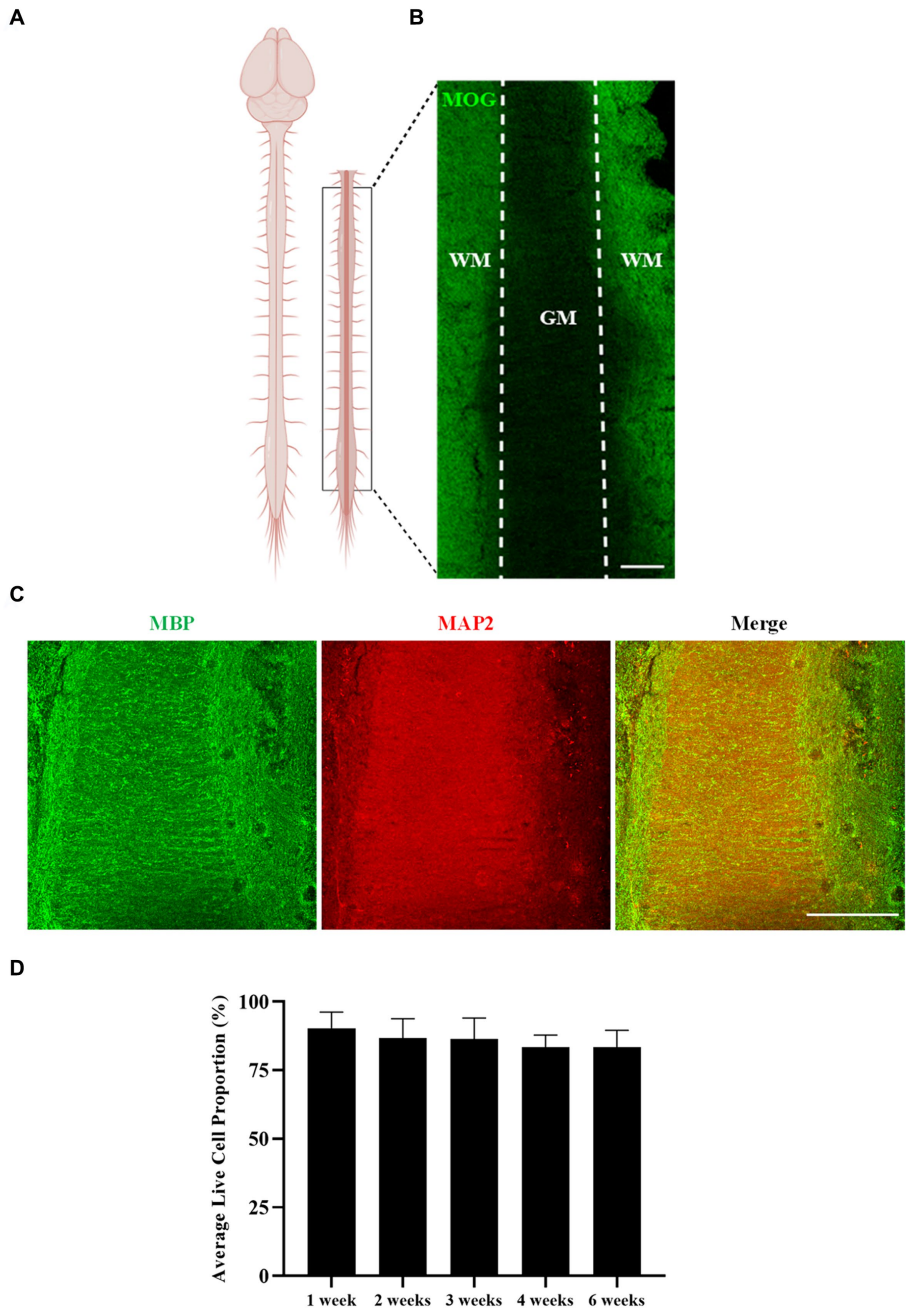
Rat spinal cord longitudinal organotypic slice cultures remain viable and are myelinated for up to 6 weeks in culture. **(A)** Diagram depicting the region/orientation of slices generated. **(B,C)** Spinal cord organotypic slices retain the gray matter (GM)/white matter (WM) boundary, as depicted by MOG, MBP, and MAP2 staining. Scale 200 μm. **(D)** Quantification of the ratio of live to dead cells in organotypic spinal cord slices over 6 weeks. Each bar represents mean ± SEM *n* = 4. No statistical significance was determined by one-way ANOVA.

Myelination of untreated longitudinal spinal cord slices was assessed after 6 weeks in culture (Figure 2). Spinal cord slices were cut longitudinally, as opposed to the common sagittal sectioning orientation, and preservation of the authentic gray matter/white matter boundary was visible in the slices (Figures 2B,C). Abundant MOG+ and MBP+ staining was seen making up the white matter tracts of the cord, with MAP2+ staining visible in the gray matter. This shows that not only is the spinal cord’s inherent architecture maintained over 6 weeks of culturing, but importantly so is myelination. Reinforcing this, quantification of the proportion of live to dead cells in the white matter region of the spinal cord slices showed sustained viability over 6 weeks of culturing, with an average live cell proportion of 86.03% ± 2.55% [*F*(4,15) = 0.333, *p* = 0.851] (Figure 2D).

These results demonstrate that rat longitudinal spinal cord slices can be cultured for up to 6 weeks *ex vivo* with no alteration in tissue viability, and with maintenance of architectural organization and preservation of myelination.

### Treatment of organotypic slices with LPS or LPC does not alter slice viability or maintenance of the gray/white matter boundary

**Figure 3:**
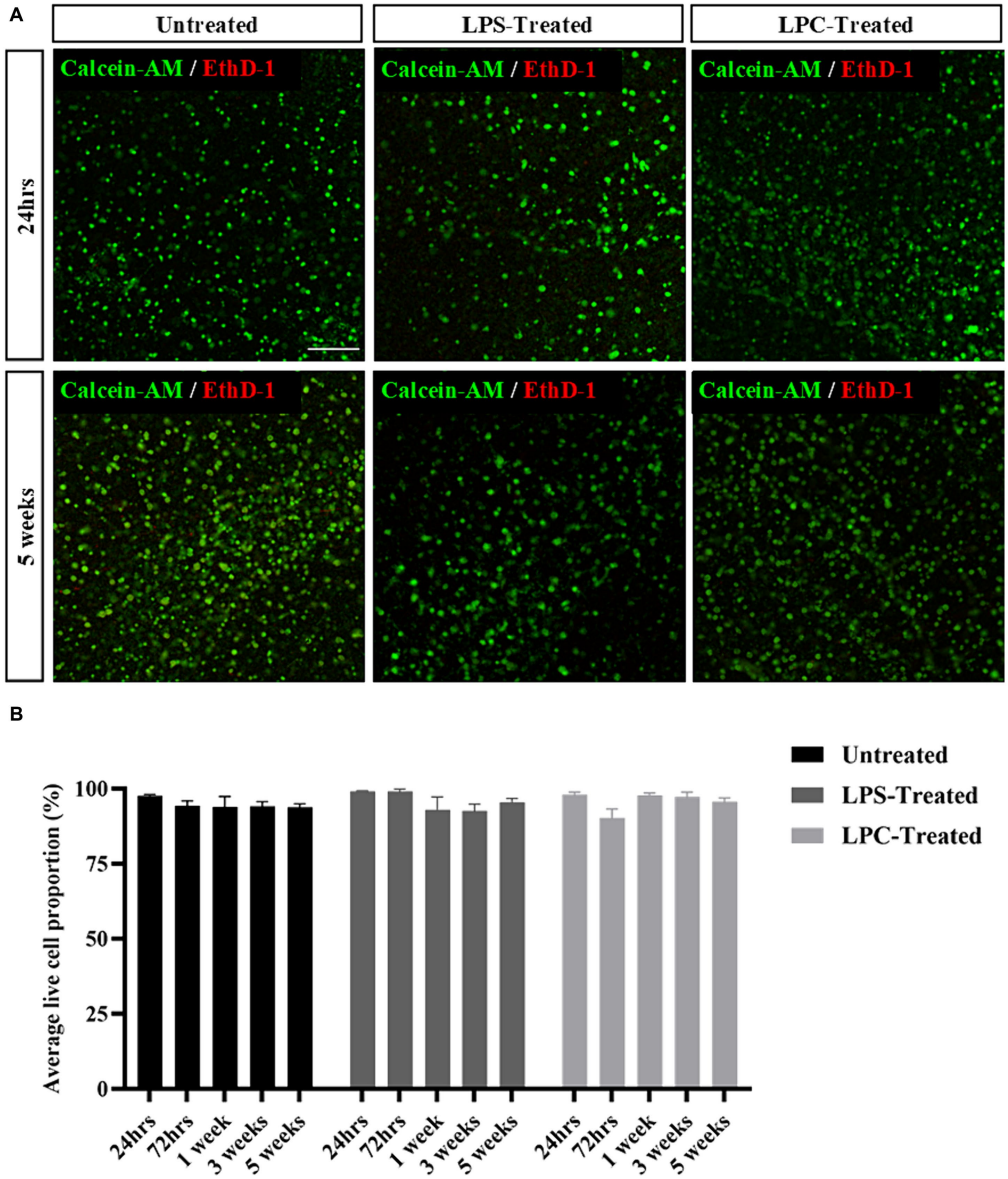
Rat organotypic slice cultures remain viable for up to 6 weeks in culture (5 weeks post-treatment) following exposure to either LPS or LPC. **(A)** Representative images of Calcein-AM+/EthD-1+ staining in untreated slices, LPS-treated and LPC-treated slices at 24 h and 5 weeks post-treatment. Scale: 200 μm. **(B)** Quantification of the ratio of live to dead cells in rat brain slices up to 5 weeks post-treatment. Data are presented as percentage of calcein AM+ live cells. Data represent mean ± SEM with *n* = 4. No statistical significance was determined by two-way ANOVA.

**Figure 4:**
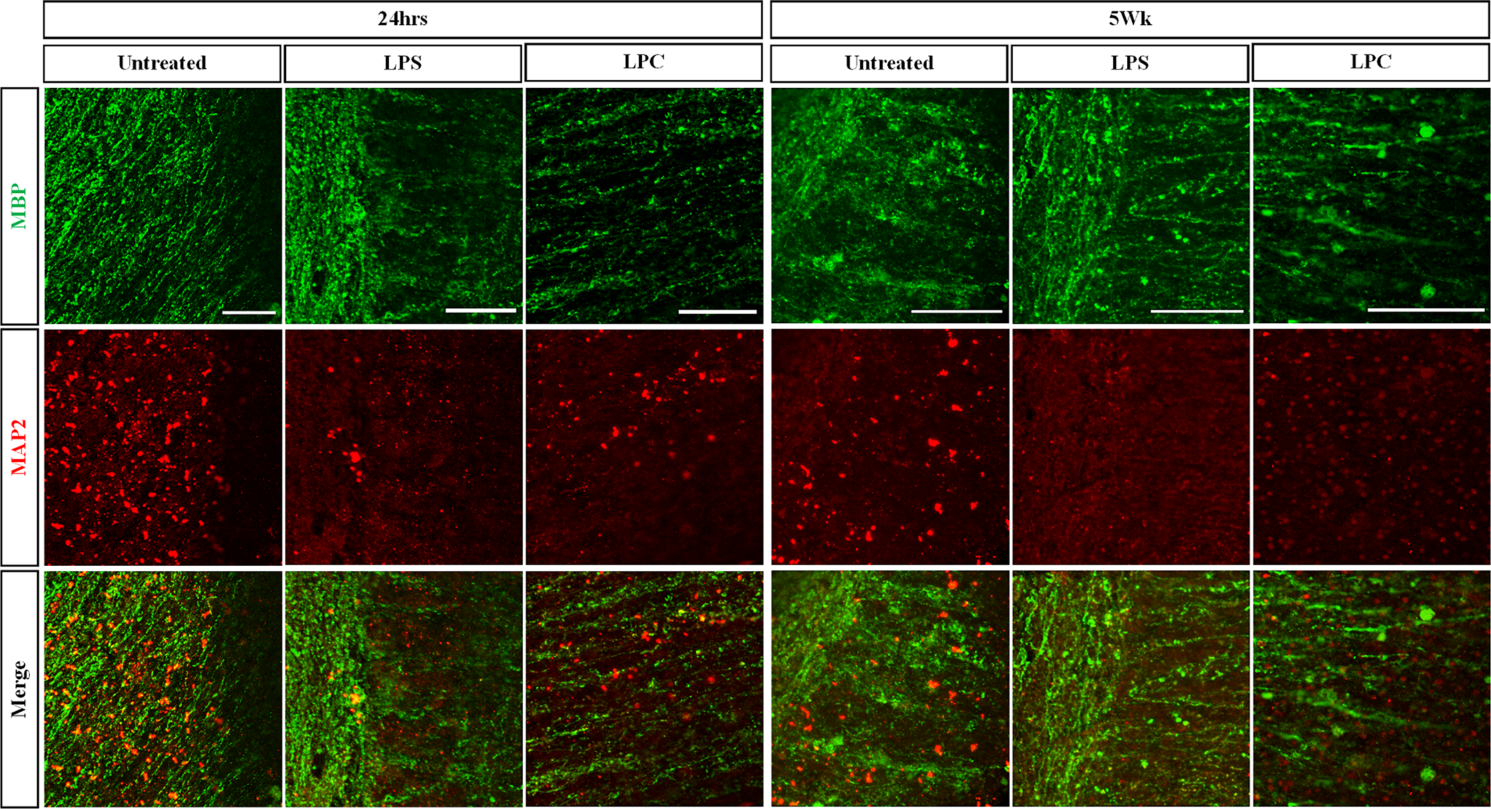
Treatment of longitudinal spinal cord organotypic slice cultures with LPS or LPC does not affect maintenance of the gray matter/white matter boundary as seen with MAP2/MBP staining. Longitudinal spinal cord slice cultures at 24 h and 5 weeks post-treatment were co-stained for the myelin marker MBP and neuronal MAP2 to assess dendritic sparing. Scale 100 μm.

Treatment of organotypic slices with LPS or LPC did not result in any visible damage, shrinkage, swelling or other macroscopic changes in the appearance of the tissue. Cell viability was assessed in the white matter of longitudinal spinal cord slices at 24 h, 72 h, 1 week, 3 weeks and 5 weeks post-treatment with either 15 μg/mL LPS or 0.5 mg/mL LPC and in corresponding untreated slices. Abundant staining of the live cell marker, calcein AM, was seen in all slices and at all time points, with few EthD-1+ dead cells present (Figure 3A). Quantification of the proportion of calcein AM+ cells demonstrated that neither treatment with LPS nor LPC altered the viability of the slices when compared to untreated slices and that the slices remained viable for up to 6 weeks in culture (5 weeks post-treatment), as seen by no effect of treatment [*F*(2,45) = 0.384, *p* = 0.683], nor time [*F*(4,45) = 1.396, *p* = 0.251] and no significant interaction between treatment and time in culture [*F*(8,45) = 1.512, *p* = 0.180] (Figure 3B). To ensure maintenance of the gray/white matter boundary with treatment of LPS or LPC, slices were co-stained with neuronal marker MAP2 and myelin marker MBP. At 24 h post-treatment, MBP and MAP2 expression is seen in all treatment groups which remains after 5-weeks post-treatment (Figure 4). This shows that neither a 24-h exposure to 15 μg/mL LPS nor a 17-h exposure to 0.5 mg/mL LPC was cytotoxic to organotypic slice cultures.

### Treatment of longitudinal spinal cord organotypic slices with LPC induces robust demyelination followed by a degree of remyelination not seen upon treatment with LPS

**Figure 5:**
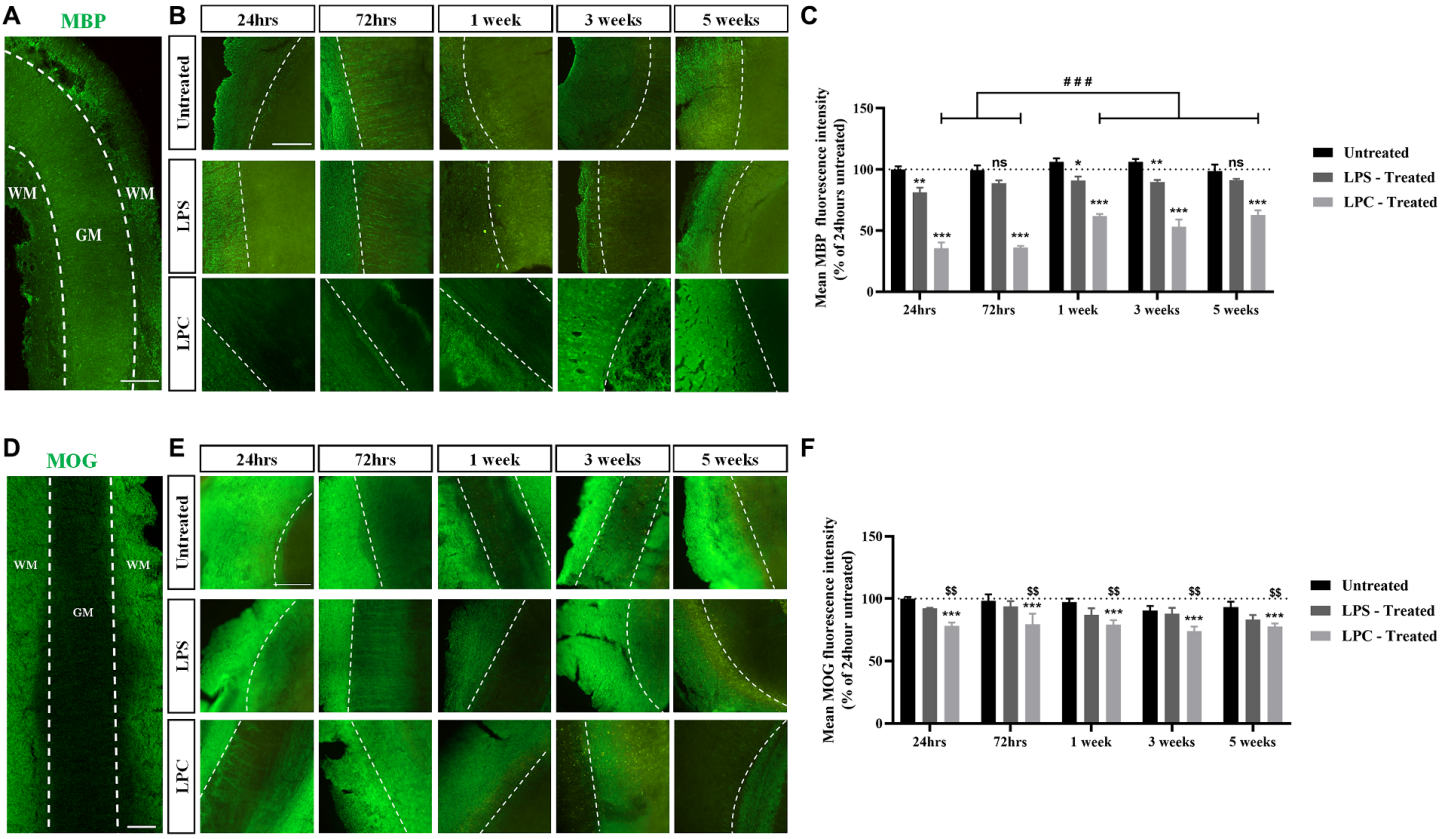
Treatment of longitudinal spinal cord organotypic slices with LPC induces robust demyelination, not seen following exposure to LPS. **(A)** MBP staining in longitudinal spinal cord slice outlining the boundary between the white matter (WM) and gray matter (GM). Scale: 200 μm. **(B)** MBP staining at weekly time points, up to 5 weeks post-treatment in untreated, LPS-treated and LPC-treated spinal cord slices. All images are oriented with WM on the left-hand side of the dotted line. Scale: 200 μm. **(C)** Quantification of the mean MBP fluorescence intensity up to 5 weeks post-treatment in untreated, LPS-treated and LPC-treated spinal cord slices. Data are presented as a percentage of MBP expression in untreated slices after 24 h. Data represent mean ± SEM with *n* = 3. Statistical significance was determined by two-way ANOVA with a significant interaction between time and treatment and pairwise comparisons performed using Bonferroni’s *post hoc* test, with ns for *p* > 0.05, * for *p* < 0.05, ** for *p* < 0.01 and *** for *p* < 0.001 indicating a significant effect of LPS treatment when compared to untreated slices and a significant effect of LPC treatment compared to both untreated and LPS-treated slices. A significant effect of time within the LPC treatment group is depicted as ### for *p* < 0.001. **(D)** MOG staining in longitudinal spinal cord slice outlining the boundary between the white matter (WM) and gray matter (GM). Scale: 200 μm. **(E)** MOG staining at weekly time points, up to 5 weeks post-treatment in untreated, LPS-treated and LPC-treated spinal cord slices. All images are oriented with WM on the left-hand side of the dotted line Scale: 200 μm. **(F)** Quantification of the mean MOG fluorescence intensity up to 5 weeks post-treatment in untreated, LPS-treated and LPC-treated spinal cord slices. Data are presented as a percentage of MOG expression in untreated slices after 24 h. Data represent mean ± SEM with *n* = 3. Statistical significance was determined by two-way ANOVA with a significant effect of treatment and pairwise comparisons performed using the Bonferroni’s *post hoc* test, with *** for *p* < 0.001 for a significant difference between LPC treatment and untreated slices and $$ for *p* < 0.01 for a significant difference between LPC treatment and LPS-treated slices.

To assess the effect of treatment with either 15 μg/mL LPS or 0.5 mg/mL LPC on myelination within the longitudinal spinal cord slice cultures, expression of MBP and MOG was examined and quantified over 6 weeks of culturing (5 weeks post-treatment). While abundant MBP staining was seen in both untreated slices and LPS-treated slices over 6 weeks of culturing, the intensity of MBP staining reduced in LPC-treated slices (Figures 5A,B). Quantification of MBP+ fluorescence intensity revealed a significant interaction between treatment and time [*F*(8,30) = 2.634, *p* = 0.026] (Figure 5C). Subsequent *post hoc* analysis demonstrated a significant reduction in mean MBP expression in LPC-treated slices at all time points, when compared to both untreated and LPS-treated slices (35.50% ± 4.85% in LPC-treated slices at 24 h and 62.57% ± 4.05% in LPC-treated slices at 5 weeks post-treatment, *p* = 0.00000000000595–0.000031), while treatment with LPS only induced a significant decrease in mean MBP fluorescence intensity at 24 h (81.23% ± 3.90%, *p* = 0.003), 1 week (90.88% ± 3.27%, *p* = 0.015) and 3 weeks post-treatment (89.83% ± 1.59%, *p* = 0.009) when compared to untreated slices (Figure 5C). Furthermore, within LPC-treated slices only, there was a significant increase in mean MBP fluorescence intensity 1, 3 and 5 weeks post-treatment (1 week: 61.88% ± 1.74%, 3 weeks: 53.22% ± 5.86%, 5 weeks: 62.57% ± 4.05%) when compared to 24 and 72 h post-treatment (24 h: 35.50% ± 4.85%, 72 h: 36.22% ± 1.17%, *p* = 0.000077–0.000107), although the mean MBP expression at the later timepoints remained significantly decrease when compared to corresponding untreated and LPS-treated slices (Figure 5C).

Analysis of MOG expression in the spinal cord slices revealed that abundant MOG staining was seen in untreated and LPS-treated slices across the time course with MOG expression present in LPC-treated albeit at a potentially lower level of expression (Figures 5D,E). Quantification of MOG+ fluorescence intensity showed a significant effect of treatment only [*F*(2,30) = 15.436, *p* = 0.000025], with no effect of time [*F*(4,30) = 1.043, *p* = 0.402], nor an interaction between time and treatment [*F*(8,30) = 0.209, *p* = 0.987] (Figure 5F). *Post hoc* analysis indicated that treatment of spinal cord slices with LPS had no effect on mean MOG+ fluorescence intensity when compared to untreated slices (*p* = 0.123), while treatment with LPC significantly reduced the mean MOG expression compared to untreated slices (*p* = 0.000017) and when compared to LPS-treated slices (*p* = 0.006) (Figure 5F).

Combined, these results indicate that treatment of longitudinal spinal cord slices with 0.5 mg/mL LPC for 17 h induces sustained demyelination with a degree of endogenous remyelination, while a 24-h exposure to 15 μg/mL LPS did not result in robust demyelination in the slices.

### Treatment of longitudinal spinal cord organotypic slices with LPS or LPC does not alter GFAP expression in the slices nor have an effect on GFAP+ cell morphology except for a reduction in total process length

**Figure 6:**
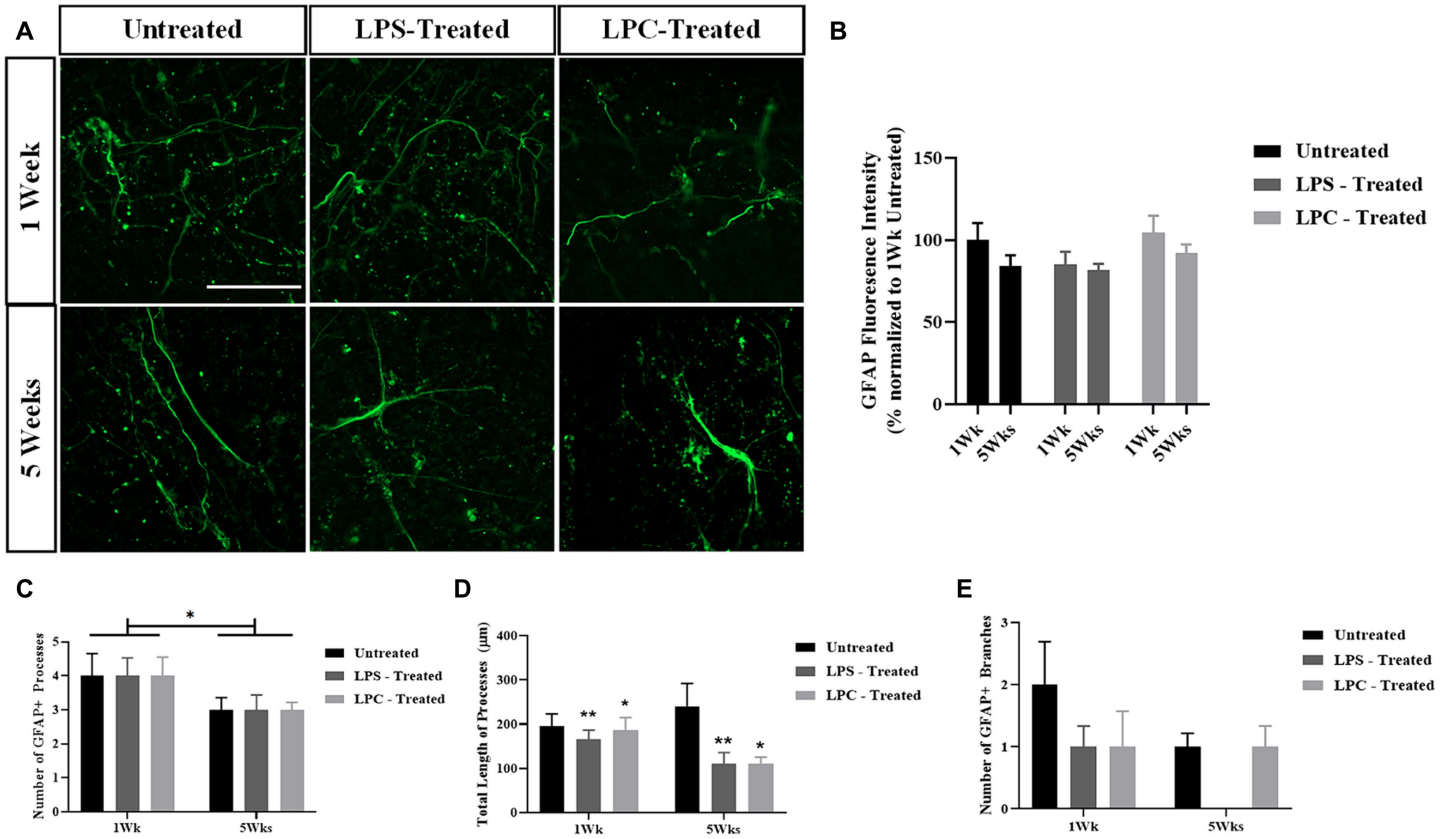
The astrocytic response to LPS or LPC treatment in longitudinal spinal cord slice cultures. **(A)** GFAP staining in longitudinal spinal cord slice cultures at 1-week and 5-weeks post-treatment in untreated, LPS-treated and LPC-treated slices. Scale 50 μm. **(B)** Quantification of GFAP fluorescence intensity at 1-week and 5-weeks post-treatment. Data are presented as a percentage of GFAP expression in untreated slices at 1-week. Data represents mean ± SEM with *n* = 3. No statistical significance was determined by a two-way ANOVA. **(C)** Number of GFAP+ processes were counted in NeuronJ. Data represents mean ± SEM with *n* = 4–7. Statistical significance was determined by two-way ANOVA with a significant effect of time and pairwise comparisons performed using the Bonferroni’s *post hoc* test, with * for *p* < 0.05. **(D)** Total length of GFAP+ processes were measured in NeuronJ. Data represents mean ± SEM with *n* = 4–7. Statistical significance was determined by two-way ANOVA with a significant effect of treatment and pairwise comparisons performed using the Bonferroni’s *post hoc* test, with * for *p* < 0.05 and ** for *p* < 0.01. **(E)** Number of GFAP+ branches were counted in NeuronJ. Data represents mean ± SEM with *n* = 4–7. No statistical significance was determined by two-way ANOVA.

To assess the effect of 15 μg/mL LPS or 0.5 mg/mL LPC treatment on astrocyte activation in longitudinal spinal cord slice cultures, untreated, LPS and LPC-treated slices were stained for the astrocyte marker glial fibrillary acidic protein (GFAP) at 1 week and 5 weeks post-treatment. GFAP expression was observed in all slices at each timepoint and treatment group (Figure 6A). Quantification of GFAP+ fluorescence intensity demonstrated no significant change in the expression of GFAP within slices regardless of treatment or time (Figure 6B), as seen by no effect of treatment [*F*(2,12) = 0.327, *p* = 0.727], nor time [*F*(1,12) = 0.514, *p* = 0.487] and no significant interaction between treatment and time in culture [*F*(2,12) = 0.063, *p* = 0.939].

To assess potential changes in astrocytic morphology in response to LPS or LPC treatment, the morphology of individual astrocytes was analyzed, quantifying the number of GFAP+ processes, the total length of the processes and the number of GFAP+ branches (Figures 6C–E). Treatment with LPS or LPC had no effect on the number of GFAP+ processes [*F*(2,27) = 0.175, *p* = 0.840] nor on the number of GFAP+ branches [*F*(2,27) = 0.697, *p* = 0.507], but did induce a reduction in total process length, irrespective of time [*F*(2,27) = 4.501, *p* = 0.021 with *p* = 0.009 for LPS compared to untreated and *p* = 0.024 for LPC compared to untreated following pairwise comparisons]. The number of GFAP+ processes was seen to reduce from ~4 on average at 1 week to ~3 on average at 5 weeks across all treatments [*F*(1,27) = 5.774, *p* = 0.023 for an effect of time only].

Overall, treatment with LPS or LPC did not alter GFAP expression in the slices and only altered the total length of the GFAP+ processes while not affecting the number of processes and branches.

## Discussion

Pre-clinical models of demyelination have been invaluable in understanding underlying cellular and molecular mechanisms, and in the development of remyelination therapies. However, most studies assess demyelination within the context of the brain, in particular the corpus callosum, and do not investigate demyelination within the spinal cord, which is relevant in both MS and SCIs. To address this, we investigated the use of longitudinal spinal cord slice cultures to model demyelination *ex vivo*. We demonstrate that rat spinal cord slice cultures generated via longitudinal sectioning remain viable for up to 6 weeks in culture with retention of the anatomical gray matter/white matter boundary as evident by MBP and MOG staining of the white matter and neuronal MAP2 staining of the central gray matter. This is an important finding demonstrating the retention of long white-matter tracts surrounding gray matter which had previously been suggested to degenerate in *ex vivo* systems (Kim et al., 2010). Additionally, the continued viability for up to 6 weeks in culture is an advancement of our system, as to the best of our knowledge, no other spinal cord organotypic slice cultures have reported viability beyond 4 weeks in culture (Bonnici and Kapfhammer, 2008; Sypecka et al., 2015; Liu et al., 2017). Our spinal cord *ex vivo* system thus provides a novel platform for long-term studies of the spinal cord.

Modeling demyelination within an *ex vivo* system is a relatively well-established technique, however previous studies have focused primarily on cerebellar tissue. This is important to consider as there are key cellular composition differences between the cortex, cerebellum, hippocampus, and spinal cord with studies suggesting hippocampal and cerebellar cells to be more sensitive to oxidative stress, a key factor involved in the process of demyelination (Wang and Michaelis, 2010). To model demyelination within the spinal cord, we used two compounds, LPC and LPS, which have previously been used to induce demyelination *ex vivo* on cerebellar slice cultures (Birgbauer et al., 2004; Di Penta et al., 2013).

Treatment of longitudinal spinal cord slices with 0.5 mg/mL LPC for 17 h induced robust demyelination, reducing MBP expression to ~35% of control untreated slices after 24 h. Furthermore, 1-week post-treatment MBP expression was restored back to ~61% of control untreated slices indicative of incomplete endogenous remyelination. We also observed a reduction in the expression of the minor myelin component MOG to ~78% of control untreated slices 24 h post-treatment and this did not change over subsequent 5 weeks of culturing. Our findings align with those of Birgbauer et al. (2004) who treated cerebellar slices with LPC and saw a qualitative reduction in MBP and MOG staining 24 h post-treatment, although this was not quantified. To quantify the extent of demyelination, Birgbauer et al. (2004) undertook a 2′,3’-Cyclic-nucleotide 3′-phosphodiesterase (CNPase) assay using HPLC and reported a 40–50% reduction in CNPase activity 24-48 h post-LPC when compared to control untreated slices. Interestingly, they also observed remyelination, as exhibited by the qualitative restoration of MBP expression back to baseline levels and an increase in CNPase activity at 4 days post-treatment with a further rise back to control levels by 8 days post-LPC. Although some MOG-positive processes were seen to align with axons, these were fewer than in control cerebellar slices even at 11 days post-treatment, suggesting incomplete remyelination. This highlights a different temporal response of MBP and MOG expression to LPC treatment. Despite both being components of the myelin sheath, MOG is one of the last myelin markers to be expressed by mature myelinating oligodendrocytes and hence it informs the degree of demyelination and remyelination. Indeed, in our study the relatively constant reduction in MOG expression throughout the 5 weeks of culturing post-LPC treatment paired with the greater reduction in MBP expression followed by a rise in MBP expression, indicates some sparing of mature myelinating oligodendrocytes and/or the lack of production of new mature myelinating oligodendrocytes. Overall, LPC treatment of longitudinal spinal cord slices induced demyelination followed by incomplete endogenous remyelination. Our rat longitudinal spinal cord slice culture system thereby mimics the demyelination and endogenous remyelination not only seen *in vivo* but that is also known to occur in MS and SCI, making our *ex vivo* platform a clinically relevant tool for mechanistic studies and testing of potential therapeutic strategies.

LPS is an inflammatory compound that has been shown to induce demyelination in *ex vivo* cerebellar slices (Di Penta et al., 2013). However, LPS is predominantly used in the context of neuroinflammation and neurodegeneration (Huuskonen et al., 2005; Ravikumar et al., 2012; Giacco et al., 2019). Neuroinflammation is highly prevalent in spinal cord demyelination seen in MS and SCI, hence we proposed LPS could be effective at inducing spinal cord demyelination. Following treatment of longitudinal spinal cord slices with 15 μg/mL LPS, we observed an initial reduction in MBP expression to ~81% of control untreated slices which was not sustained for 5 weeks post-treatment. We also saw no effect on MOG expression. This contrasts with Di Penta et al. (2013) who treated cerebellar slices with LPS and reported demyelination as measured through a reduction in CNPase activity and MBP immunostaining at 24 h post-treatment which was sustained to 96 h post-LPS. As an inflammatory compound, LPS is known to activate microglia and through this mechanism is thought to contribute to demyelination (Li et al., 2008). Therefore, the use of spinal cord tissue rather than cerebellar tissue with a different composition of cells may be a reason for these conflicting results.

Demyelination is coupled to inflammatory processes well documented to involve the activation of resident astrocytes and microglia recognized by changes in cell number and morphology (Traiffort et al., 2020). To assess changes in astrocyte activation, longitudinal spinal cord slices were treated with LPC or LPS and stained for the astrocytic marker GFAP. We found that treatment had no effect on overall GFAP expression at 1-week or 5-weeks post-treatment. This finding is in line with Giacco et al. (2019) who reported no change in either Iba1 or GFAP staining intensity in spinal cord slices following a 6 h treatment with 1 μg/mL LPS. Interestingly, this study reported increases in IbA1 and GFAP staining intensity in response to cytokine stimulation, suggesting the potential need of cytokine treatment in combination with LPS or LPC to induce astrocytic and microglial activation alongside demyelination, which has also been demonstrated in hippocampal slices (Cammarota and Boscia, 2023). LPS is considered a pro-inflammatory compound, however, LPC is a glial toxin and hence only considered to indirectly increase GFAP expression. Indeed, Yoon et al. (2017) demonstrated an increase in GFAP staining intensity following an *in vivo* LPC insult, however, this was delivered as a focal insult as opposed to diffuse injury such as in our *ex vivo* system, which could explain the unchanged GFAP intensity we observed.

GFAP+ astrocytes undergo morphological changes as an indication of activation in response to inflammatory insults. To investigate this, processes analysis on individual GFAP+ astrocytes present in untreated, LPS-treated and LPC-treated slices was performed. We found no change in the number of GFAP+ processes in response to treatment, however, saw a reduction in the number of processes between 1 week and 5 weeks post-treatment across all treatments. We likewise saw no change in the number of branches between treatments but observed a reduction in the total length of GFAP+ processes in response to both LPC and LPS treatment irrespective of time. It has previously been shown in hippocampal slices that GFAP+ astrocytes initially appear with star-shaped morphology but throughout culture undergo morphological changes to more fibrous-like cells, extending out long processes (Huuskonen et al., 2005). The reduction in total process length seen with LPC and LPS treatment suggests divergence from this response which suggests a treatment effect.

Taken together, neither LPS nor LPC treatment had an effect on GFAP+ expression nor on GFAP morphology except for a reduction in total process length. It has been suggested that IbA1+ microglia display a greater response to LPS in *ex vivo* systems, however, we could not assess this in our system as the IbA1 antibodies were incompatible with iDISCO optical clearing.

Overall, we have demonstrated that rat longitudinal spinal cord slice cultures remain viable for up to 6 weeks in culture with retention of key cytoarchitectural features. We have shown that rat longitudinal spinal cord slices can be used to model demyelination through a single 17 h exposure to 0.5 mg/mL LPC, a treatment that induces robust demyelination followed by spontaneous incomplete remyelination which mimics what takes place *in vivo*. While studies have suggested LPS as a demyelinating compound, 24 h exposure to 15 μg/mL LPS was insufficient in inducing robust demyelination in spinal cord slices. This may be due to the different cellular compositions between the spinal cord and the cerebellum in terms of the presence of microglia and highlights the importance of studying and modeling demyelination within the spinal cord itself. LPC-demyelinated rat longitudinal spinal cord organotypic slices will provide a novel *ex vivo* platform for future longitudinal studies investigating the cellular and molecular mechanisms underlying demyelination and endogenous remyelination, and for the testing of potential therapeutic strategies including cell-based remyelination therapies for MS and SCI.

## Data availability statement

The original contributions presented in the study are included in the article/supplementary material, further inquiries can be directed to the corresponding author.

## Ethics statement

The animal study was approved by the University of Auckland Animal Ethics Committee. The study was conducted in accordance with the local legislation and institutional requirements.

## Author contributions

BH: Formal analysis, Investigation, Methodology, Writing – original draft. MD: Formal analysis, Investigation, Methodology, Writing – review & editing. BC: Conceptualization, Writing – review & editing. AM-C: Conceptualization, Funding acquisition, Methodology, Supervision, Writing – review & editing.
